# Characteristics of Gamblers Who Use the French National Problem Gambling Helpline and Real-Time Chat Facility: Longitudinal Observational Study

**DOI:** 10.2196/13388

**Published:** 2020-05-20

**Authors:** Stéphane Darbeda, Henri-Jean Aubin, Michel Lejoyeux, Amandine Luquiens

**Affiliations:** 1 Addiction Science Paris Sud University CESP Inserm UMR-1018 Villejuif France; 2 ED3C University Pierre And Marie Curie Paris France; 3 Department of Psychiatry and Addiction Medicine Hospitals Paris Nord Val de Seine, Assitance Publique Hôpitaux de Paris Paris Diderot University Paris France; 4 Department of Addiction Science Paris Sud University CESP-Inserm UMR-1018 Villejuif France; 5 Department of Addiction Medicine University Hospitals Paris Sud, Assistance Publique Hôpitaux de Paris Villejuif France; 6 Centre de Mathématiques Appliquées École Polytechnique Palaiseau France

**Keywords:** gambling, helpline, chat, counseling, gender, time-series analysis

## Abstract

**Background:**

Problem gambling is a growing public health issue that is characterized by low rates of face-to-face help seeking. Helplines and real-time chat services could reduce shortfalls in treatment.

**Objective:**

This study aimed to (1) describe the characteristics of gamblers contacting a government-funded help service, (2) study the evolution of their characteristics over time, (3) evaluate the differences between subgroups (ie, gender, media used for gambling, and media used to contact the service), and (4) explore factors influencing referral to care.

**Methods:**

From January 2011 to December 2015, a government-funded gambling helpline and real-time chat website in France received 9474 contacts from gamblers. Counselors filled in a form for each contact, collecting demographics, gambling characteristics, and referrals. Time-series analyses were performed. Univariate logistic models were used to assess differences across subgroups. A multivariate analysis was conducted to determine the variables related to an actual referral.

**Results:**

Gamblers were predominantly men (7017/9474, 74.07%); the average age was 41 years (SD 14). Compared with the men, the women were older (mean 50.7 years, SD 14.0 vs mean 37.9 years, SD 13.0, respectively; *P*<.001), were more often solely offline gamblers (1922/2457, 78.23% vs 4386/7017, 62.51%, respectively; *P*<.001), and had different gambling patterns. Compared with helpline contacts, real-time chat contacts were more often men (124/150, 82.7% vs 3643/4881, 74.64%, respectively; *P*=.04), younger (mean 32.8 years, SD 12.9 vs mean 41.3 years, SD 14.3, respectively; *P*<.001), more often poker gamblers (41/150, 27.3% vs 592/4881, 12.13%, respectively; *P*<.001), and more often web-based gamblers (83/150, 55.3% vs 1462/4881, 29.95%, respectively; *P*<.001). Referral was positively associated with betting (adjusted odds ratio [aOR] 1.46, 95% CI 1.27-1.67; *P*<.001), casino gambling (aOR 1.38, 95% CI 1.21-1.57; *P*<.001), scratch cards (aOR 1.83, 95% CI 1.58-2.12; *P*<.001), poker gambling (aOR 1.35, 95% CI 1.14-1.61; *P*<.001), lottery (aOR 1.27, 95% CI 1.03-1.56; *P*=.03), weekly gambling (aOR 1.73, 95% CI 1.40-2.15; *P*<.001), request for referral (aOR 17.76, 95% CI 14.92-21.13; *P*<.001), and a history of suicide attempts (aOR 2.13, 95% CI 1.51-3.02; *P*<.001), and it was negatively associated with web-based gambling (aOR 0.86, 95% CI 0.75-0.98; *P*=.030) and refusal to be referred (aOR 0.35, 95% CI 0.26-0.49; *P*<.001).

**Conclusions:**

The governmental helpline and chat contacts included a broad range of sociodemographic profiles. Compared with the helpline, real-time chat exchanges reached a younger population of web-based gamblers, which was the target population. The development of the gambling helpline and help online website is a considerable challenge for the future.

## Introduction

### Background

Problem gambling is a growing public health issue. A recent meta-analysis showed that the prevalence rate of problem gambling in the past year in the general population ranged from 0.12% to 5.8% worldwide and from 0.12% to 3.4% in Europe [1]. In France, the prevalence rate of problem gambling increased from 1.3% in 2010 to 1.9% in 2014 [2,3]. Problem gambling is characterized by low levels of help seeking, estimated at 7% to 29% depending on the country [3-9]. The barriers identified to help seeking are (1) intention to handle gambling problems on one’s own, (2) stigma and minimization of problems, (3) concerns about treatment content and quality, (4) lack of knowledge about treatment availability, and (5) practical issues of attending treatment [10]. One study provided evidence that help seeking occurred most often when gambling-related harm had become significant, especially financial problems, relationship issues, and negative emotions [11]. Problem gamblers often seek help after they have run out of other options [12].

Helplines have been used for many years in different settings, such as suicidal crisis [13], cancer [14], chronic rheumatic diseases [15], eating disorders [16], or substance use disorders [17]. Helplines and real-time chat for problem gambling have been implemented and assessed in several countries [18-25]. The severity of problem gambling has often been found to be considerable, with high rates of suicidal thoughts observed among helpline contacts [21-23]. Helpline callers have been consistently reported to be heterogeneous groups, with gender differences [19,20,22,25-27]. Most studies on gambling helplines users have reported characteristics close to those known in the broader population of problem gamblers, with more men [19,20,24-26], and gambling types following the commercial offer at the time of the study [18,24,28]. Contacts with helplines have been shown to concern mostly first-time treatment seekers [19,23]. Contacts could thus belong to a population with serious problems [22,23,26] and uncertain demands, and to motivate them and provide them with the most appropriate help could therefore be particularly important. In 2011, more than three-fourths of the contacts to the Problem Gamblers Help Network of West Virginia, who were offered guidance, agreed to be referred [23]. Active referral could be particularly worthwhile in this otherwise untreated population.

In France, in May 1990, on proposal by the *Mission interministérielle de lutte contre les drogues et les conduites addictives*, the government adopted a plan to establish a national telephone information and prevention service on drugs and drug addiction under the supervision of *Addictions Drogues Alcool Info Service* (ADALIS). Since 2010, the Web-based gambling market has been open to competition and is being regulated by the law of May 12, 2010. In June 2010, the telephone helpline for problem gambling *Joueurs Info Service* (JIS) was implemented, followed by the real-time chat website in May 2013. Throughout this study, the term “contact” refers to any contact with the JIS and does not distinguish between phone calls to the helpline and the use of real-time chat. The medium is specified whenever necessary.

### Objectives

The purpose of this study was to describe the characteristics of gamblers who contacted the service over the study period and to compare them in subgroups according to gender, the media used for gambling (solely offline or Web-based gamblers), and the channel used to contact the service (helpline callers or real-time chat users). It seemed important to study the evolution of characteristics of the population over time just after the opening of the Web-based gambling market to competition. We also explored factors associated with referral to care. As a result of the opening of the Web-based gambling market to competition, we were expecting an increase in calls concerning Web-based gambling, from younger gamblers, and more men [29,30]. In reference to the international literature on problem gambling helplines and recent gambling prevalence studies in France, we hypothesized that men and women, as well as helpline and real-time chat users, would present different characteristics and gambling patterns. Specifically, we expected men to be younger than women [19,20,22,25], men to be more frequently engaged in poker gambling and bets [3,19,25,31], and women to gamble more frequently in a casino [18,19,25]. The target population of real-time chat was young and Web-based gamblers, so we expected real-time chat users to be younger and to use Web-based gambling more frequently than helpline callers. In addition, we expected that barriers [10] and motivators [11] for help seeking would appear as variables influencing the actual referral of the contacts.

## Methods

### Description of the Helpline

Since June 2010, under the supervision of the Ministry of Health, JIS has been a free, national, remote support service for problem gambling. JIS is based on the rules of anonymity, confidentiality, neutrality, and absence of moral judgment. Its missions are to inform, advise, support, and guide contacts. All the helpline and website staff are salaried and have received initial training, the aim of which was to develop listening skills and availability and to acquire the necessary knowledge. The helpline is accessible from 7:00 AM to 2:00 AM. Since May 2013, the JIS has also offered real-time chat enabling online individual interviews. No follow-up is offered following the contact. Referral is offered at the discretion of the counselors.

### Population

**Figure 1:**
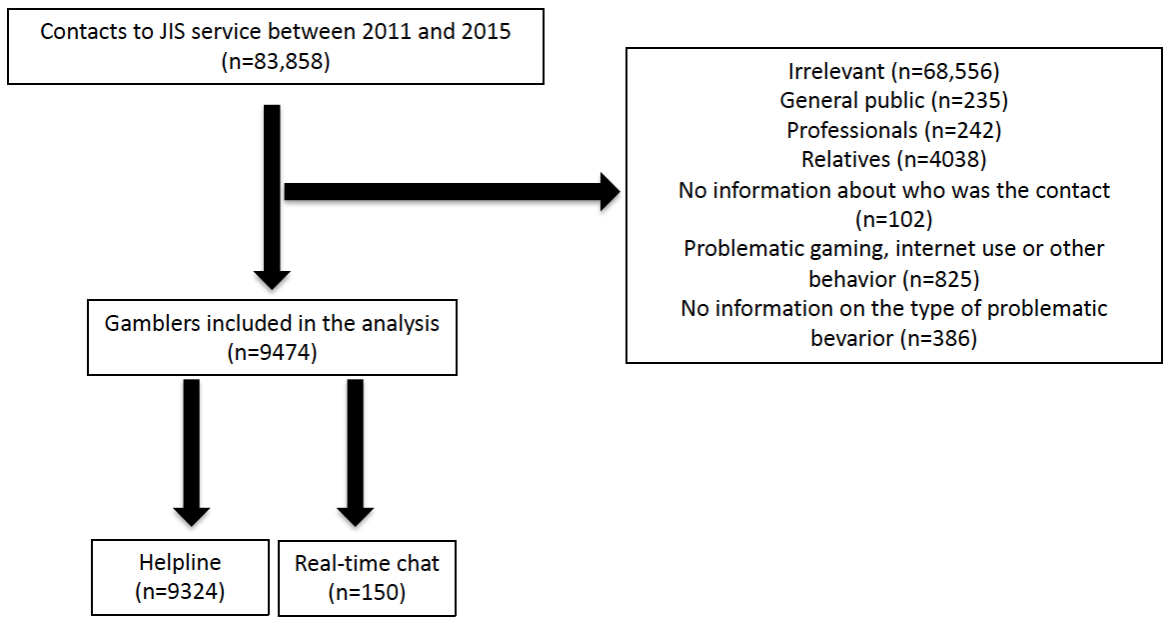
A flow diagram. JIS: Joueurs Info Service.

Ethical approval (N°2018-031) was obtained from the institutional review board of Paris Diderot University Hospitals (IRB 00006477). From the JIS database, several inclusion criteria were defined for contacts: (1) all contacts between 2011 and 2015, (2) contacts considered to be relevant to the purpose of JIS, (3) contacts from gamblers and not from gamblers’ relatives, and (4) contacts concerned gambling and not any other problem behavior. Between January 2011 and December 2015, the helpline received 83,858 contacts, of which 68,556 (81.75%) were considered irrelevant, ie, errors or jokes. From the remaining 15,302 contacts related to the purpose of JIS, 4038 (26.4%) contacts from gamblers’ relatives were excluded, and we included 9474 (61.9%) contacts who were gamblers (Figure 1).

### Data Collection

For each contact, the counselors systematically collected the following data: (1) medium used to contact JIS (helpline or real-time chat); (2) characteristics of the contacts: age and gender, gambler or relative, and first contact with the platform; (3) gambling types: bets (included gambling on sports and horse racing, without distinction), casino, scratch cards, poker, lottery, and other games; (4) media used for gambling (ie, solely offline, Web-based, or both); (5) proxies for the severity of problem gambling: gambling frequency (less than weekly vs weekly or more), time since gambling initiation, and history of suicide attempts; (6) contacts’ attitudes to referral (request for referral or refusal to be referred); (7) actual referral (we considered that the contact led to an actual referral when he or she was encouraged to resume a follow-up that already started or to consult a known or a new address). We considered that the contact was not referred when he/she was redirected to websites or helplines related to ADALIS or when he/she was not offered a referral; (8) self-exclusion. In France, self-exclusions related to casino venues and Web-based gambling run for 3 years. Self-excluded gamblers are not accompanied by any type of medical or social counseling.

Missing Data

Two variables—medium used and duration of the contact—were automatically collected and, therefore, presented no missing data. Age, media used for gambling, gambling frequency, and time since gambling initiation had high rates of missing data (32.4%, 34.0%, 50.2%, and 59.3%, respectively), while gender was collected in almost all cases (0.3% missing data). Contact referral was systematically reported and presented no missing data. The main reason for missing data was when the counselor failed to ask the question, which can be considered missing at random (MAR) [32]. Multiple imputation is a general approach to the problem of missing data, which aims to allow for the uncertainty generated by the missing data by creating several different plausible imputed datasets—using existing values from other variables—and appropriately combining results obtained from each of them. Multiple imputation should be used for MAR data, but multiple imputation can produce more accurate estimates than a complete case analysis even when MAR assumptions are not met [33]. Missing data were imputed by multiple imputation using chained equations, and the package “mice” version 3.3.0 [34] under R, version 3.4.4, was used for the imputation.

### Data Analysis

For the time-series analyses, univariate linear regressions—based on multiple imputed data—were calculated to assess the relationship between the different variables and time. Univariate logistic regression analyses based on multiple imputed data were completed for each of the categories of independent variables to determine relationships with each dichotomous dependent variable (male vs female gender, solely offline vs Web-based or both, and helpline vs real-time chat users). A multivariate logistic model was constructed from the univariate model using the stepwise method with backward elimination to analyze the actual referral of contacts. Independent variables were examined for collinearity using correlation matrices before the completion of the multiple regression analysis. All statistical tests were 2-sided, with a significance threshold of 0.05. The data were analyzed using R 3.4.4. software.

## Results

### Description of Contacts

The description of contacts is shown in Table 1. Gamblers were predominantly men (7017/9474, 74.07%), the mean age was 41.2 years (SD 14.4; IQR 30.0-51.0), with less than 3% of contacts younger than 20 years and approximately one-quarter older than 50 years. The average duration of the contact was 14.4 min, but variability was large (SD=24.6). Less than a quarter (2131/9474, 22.49%) mentioned having contacted the service previously. The main types of gambling were bets, casino, scratch cards, and poker. The majority of gamblers used solely offline gambling (6308/9474, 66.58%), just over a quarter used only Web-based gambling (2676/9474, 28.25%), and 5.17% (490/9474) used both. The average time since gambling initiation was 16 years (SD 10.2). Approximately one-quarter asked for a referral (2279/9474, 24.06%), whereas 2.30% (218/9474) refused to be referred. More than half the contacts led to a referral. Very few gamblers reported having a history of suicide attempts (193/9474, 2.04%), and 5.03% (477/9474) reported self-exclusion.

### Evolution Over Time

We observed stability over the study period for the average age of the gamblers and the average duration of the contact and for the proportions of women and weekly or more than weekly gamblers (see Multimedia Appendix 1). There was an increase in the proportion of contacts for solely offline gambling (beta=3.02; 95% CI 1.76-4.27; *P*<.001) and for certain types of gambling: casino (beta=2.20; 95% CI 1.24-3.16; *P*<.001) and scratch cards (beta=1.23; 95% CI 0.55-1.91; *P*<.001). In contrast, the proportion of contacts for bets and lottery games was stable over the period, and there was a decrease in the proportion of contacts for poker (beta=−2.35; 95% CI −2.89 to −1.82; *P*<.001) and other games (beta=−0.75; 95% CI −1.10 to −0.40; *P*<.001). Between May 2013 and December 2015, we observed an increase in the number of real-time chat users (beta=3.02; 95% CI 1.76-4.27; *P*=.005).

### Gender Differences

As shown in Table 1, men outnumbered women (7017/9474, 74.07% vs 2457/9474, 25.93%, respectively). The men were younger than the women (mean 37.9 years, SD 13.0 vs mean 50.7 years, SD 14.0, respectively; *P*<.001) and were mainly younger than 40 years (4143/7017, 59.04%), whereas the majority of women were aged ≥50 years (1387/2457, 56.45%). Contact durations were the same. There were significant differences in gambling types between men and women. Although men betted more than women (3405/7017, 48.52% vs 409/2457, 16.65%, respectively; *P*<.001) and were more frequently engaged in poker gambling (1321/7017, 18.83% vs 134/2457, 5.45%, respectively; *P*<.001), women compared with men more frequently gambled in casino games (1144/2457, 46.56% vs 1812/7017, 25.82%, respectively; *P*<.001), on scratch cards (696/2457, 28.33% vs 1170/7017, 16.67%, respectively; *P*<.001), and on lotteries (302/2457, 12.29% vs 718/7017, 10.23%, respectively; *P*=.005). Men gambled weekly more often than women (6359/7017, 90.62% vs 2125/2457, 86.49%, respectively; *P*=.001). Compared with men, women were more often solely offline gamblers (1922/2457, 78.23% vs 4386/7017, 62.51%, respectively; *P*<.001). There was no difference for history of suicide attempts. Women were more often self-excluded than men (157/2457, 6.39% vs 320/7017, 4.56%, respectively; *P*<.001). Compared with women, men more often expressed an attitude toward referral, whether in the form of a request (1798/7017, 25.62% vs 481/2457, 19.58%, respectively; *P*<.001) or refusal (176/7017, 2.51% vs 42/2457, 1.71%, respectively; *P*=.02). Men were more likely to be referred than women (3974/7017, 56.63% vs 1247/2457, 50.75%, respectively; *P*<.001).

**Table 1:** Characteristics of contacts by gender (N=9474). Univariate logistic regression analysis; the significance code lies on the side where the proportion, or the mean, is the highest.

Demographics		Total	Male	Female
Population, n (%)		9474 (100.00)	7017 (74.07)	2457 (25.93)
**Age (years), mean (SD)**		41.2 (14.4)	37.9 (13.0)	50.7 (14.0)^a^
	<20	256 (2.70)	243 (3.46)^a^	13 (0.53)
	20-29	1995 (21.06)	1838 (26.19)^a^	157 (6.39)
	30-39	2413 (25.47)	2062 (29.39)^a^	351 (14.29)
	40-49	2093 (22.09)	1544 (22.00)	549 (22.34)^b^
	50-59	1562 (16.49)	837 (11.93)	725 (29.51)^a^
	≥60	1155 (12.19)	493 (7.03)	662 (26.94)^a^
Duration of contact (min), mean (SD)		14.4 (24.6)	14.5 (27.4)^b^	14.1 (13.8)
**Gambling type, n (%)**				
	Bets	3814 (40.26)	3405 (48.52)^a^	409 (16.65)
	Casino	2956 (31.20)	1812 (25.82)	1144 (46.56)^a^
	Scratch cards	1866 (19.70)	1170 (16.67)	696 (28.33)^a^
	Poker	1455 (15.36)	1321 (18.83)^a^	134 (5.45)
	Lottery	1020 (10.77)	718 (10.23)	302 (12.29)^c^
	Others	466 (4.92)	350 (4.99)^b^	116 (4.72)
**Media used for gambling, n (%)**				
	Solely offline	6308 (66.58)	4386 (62.51)	1922 (78.23)^a^
	Web-based or both	3166 (33.42)	2631 (37.49)^a^	535 (21.77)
**Proxies for severity of gambling**				
	Gambling frequency weekly or more, n (%)	8484 (89.55)	6359 (90.62)^c^	2125 (86.49)
	Time since gambling initiation (years), mean (SD)	16.0 (10.2)	15.7 (10.2)^b^	16.6 (10.0)
	History of suicide attempt, n (%)	193 (2.04)	135 (1.92)	58 (2.36)^b^
**Contact demand, n (%)**				
	Referral requested	2279 (24.06)	1798 (25.62)^a^	481 (19.58)
	Refusal to be referred	218 (2.30)	176 (2.51)^d^	42 (1.71)
**Actual referral, n (%)**				
	Referred	5221 (55.11)	3974 (56.63)^a^	1247 (50.75)
	Nonreferred	4253 (44.89)	3043 (43.37)	1210 (49.25)^a^
Self-exclusion, n (%)		477 (5.03)	320 (4.46)	157 (6.39)^a^

^a^*P*<.001.

^b^Not significant.

^c^*P*<.01.

^d^*P*<.05.

### Differences Between Solely Offline and Other Gamblers

As shown in Table 2, solely offline gamblers were more likely to be women than Web-based gamblers (1922/6308, 30.5% vs 535/3166, 16.9%, respectively; *P*<.001). Solely offline gamblers were older than Web-based gamblers (mean 43.7 years, SD 14.5 vs mean 36.2 years, SD 12.8, respectively; *P*<.001); the majority were aged ≥40 years (3697/6308, 58.61%), whereas Web-based gamblers were mainly aged younger than 40 years (2053/3166, 64.85%). There were significant differences in gambling type between solely offline and Web-based gamblers. Although solely offline gamblers, compared with Web-based gamblers, were more often engaged in casino gambling (2367/6308, 37.52% vs 589/3166, 18.60%, respectively; *P*<.001), scratch cards (1678/6308, 26.60% vs 188/3166, 5.94%, respectively; *P*<.001), and lotteries (745/6308, 11.81% vs 275/3166, 8.69%, respectively; *P*<.001). Web-based gamblers, compared with solely offline gamblers, were more often engaged in betting (1556/3166, 49.15% vs 2258/6308, 35.80%, respectively; *P*<.001), poker gambling (1138/3166, 35.94% vs 317/6308, 5.03%, respectively; *P*<.001), and other gambling games (280/3166, 8.84% vs 186/6308, 2.95%, respectively; *P*<.001). There was no between-group difference in the proxy measures used to assess severity of gambling or for the self-exclusion rates. Solely offline gamblers, compared with Web-based gamblers, more frequently requested to be referred (1625/6308, 25.76% vs 654/3166, 20.66%, respectively; *P*<.001) and were more often actually referred (3615/6308, 57.31% vs 1606/3166, 50.73%, respectively; *P*<.001). Web-based gamblers, compared with solely offline gamblers, more often refused to be referred (93/3166, 2.94% vs 125/6308, 1.98%, respectively; *P*=.04).

### Differences Between Helpline Calls and Real-Time Chats

Between May 2013 and December 2015, chat contacts were very few (150/5031, 3.00%). As shown in Table 3, chat contacts, compared with helpline contacts, were more likely to be men (124/150, 82.7% vs 3643/4881, 74.64%, respectively; *P*=.04) and younger (mean 32.8 years, SD 12.9 vs mean 41.3 years, SD 14.3, respectively; *P*<.001). Chat contacts lasted longer than helpline contacts (mean 28.6, SD 20.3 vs mean 14.2, SD 13.9, respectively; *P*<.001). There was no between-group difference on the proxy measures of severity of gambling or for self-exclusion rates. Compared with helpline contacts, chat contacts were more often engaged in Web-based gambling (83/150, 55.3% vs 1462/4881, 29.95%, respectively; *P*<.001), poker gambling (41/150, 27.3% vs 592/4881, 12.13%, respectively; *P*<.001), and other games (16/150, 10.7% vs 182/4881, 3.73%, respectively; *P*<.001) and less often engaged in scratch card gambling (11/150, 7.3% vs 1043/4881, 21.37%, respectively; *P*<.001). Chat contacts, compared with helpline contacts, less often asked to be referred (24/150, 16.0% vs 1258/4881, 25.77%, respectively; *P*=.008), but there was no significant difference for actual referrals.

### Differences Between Referred and Nonreferred Gamblers

The multivariate logistic analysis (Table 4) enabled us to identify the following factors positively and independently associated with actual referral: betting (adjusted odds ratio, aOR 1.46, 95% CI 1.27-1.67; *P*<.001), casino gambling (aOR 1.38, 95% CI 1.21-1.57; *P*<.001), scratch cards (aOR 1.83, 95% CI 1.58-2.12; *P*<.001), poker gambling (aOR 1.35, 95% CI 1.14-1.61; *P*<.001), lottery (aOR 1.27, 95% CI 1.03-1.56; *P*=.03), gambling weekly (aOR 1.73, 95% CI 1.40-2.15; *P*<.001), referral requested (aOR 17.76, 95% CI 14.92-21.13; *P*<.001), and having a history of suicide attempts (aOR 2.13, 95% CI 1.51-3.02; *P*<.001). The following factors were negatively and independently associated with actual referral: Web-based gambling (aOR 0.86, 95% CI 0.75-0.98; *P*=.03) and refusal to be referred (aOR 0.35, 95% CI 0.26-0.49; *P*<.001).

**Table 2:** Characteristics of contacts by media used for gambling (N=9474). Univariate logistic regression analysis; the significance code lies on the side where the proportion, or the mean, is the highest.

Demographics		Solely offline	Web-based or both
Population, n (%)		6308 (66.58)	3166 (33.42)
**Gender, n (%)**			
	Male	4386 (69.53)	2631 (83.10)^a^
	Female	1922 (30.47)^a^	535 (16.90)
**Age (years), mean (SD)**		43.7 (14.5)^a^	36.2 (12.8)
	<20	113 (1.79)	143 (4.52)^a^
	20-29	1030 (16.33)	965 (30.48)^a^
	30-39	1468 (23.27)	945 (29.85)^a^
	40-49	1482 (23.49)^a^	611 (19.30)
	50-59	1248 (19.78)^a^	314 (9.92)
	≥60	967 (15.33)^a^	188 (5.94)
Duration of contact (min), mean (SD)		14.2 (22.5)	14.8 (28.5)^b^
**Gambling type**			
	Bets	2258 (35.80)	1556 (49.15)^a^
	Casino	2367 (37.52)^a^	589 (18.60)
	Scratch cards	1678 (26.60)^a^	188 (5.94)
	Poker	317 (5.03)	1138 (35.94)^a^
	Lottery	745 (11.81)^a^	275 (8.69)
	Others	186 (2.95)	280 (8.84)^a^
**Proxies for severity of gambling**			
	Gambling frequency weekly or more, n (%)	5630 (89.25)	2854 (90.15)^b^
	Time since gambling initiation (years), mean (SD)	16.7 (10.3)^b^	14.5 (9.6)
	History of suicide attempt, n (%)	131 (2.08)^b^	62 (1.96)
**Contact demand, n (%)**			
	Referral requested	1625 (25.76)^a^	654 (20.66)
	Refusal to be referred	125 (1.98)	93 (2.94)^c^
**Actual referral, n (%)**			
	Referred	3615 (57.31)^a^	1606 (50.73)
	Nonreferred	2692 (42.68)	1560 (49.27)^a^
Self-exclusion, n (%)		316 (5.01)	161 (5.09)^b^

^a^*P*<.001.

^b^Not significant.

^c^*P*<.05.

**Table 3:** Characteristics of contact who accessed the helpline and real-time chat (N=5031). Univariate logistic regression analysis; the significance code lies on the side where the proportion, or the mean, is the highest.

Demographics		Helpline	Real-time chat
Population, n (%)		4881 (97.02)	150 (2.98)
**Gender, n (%)**			
	Male	3643 (74.64)	124 (82.7)^a^
	Female	1238 (25.36)^a^	26 (17.3)
**Age (years), mean (SD)**		41.3 (14.3)^b^	32.8 (12.9)
	<20	122 (2.50)	14 (9.3)^b^
	20-29	1012 (20.73)	65 (43.3)^b^
	30-39	1283 (26.29)^c^	35 (23.3)
	40-49	1082 (22.17)^a^	17 (11.3)
	50-59	787 (16.12)^a^	12 (8.0)
	≥60 or older	595 (12.19)^a^	7 (4.7)
Duration of contact (min), mean (SD)		14.2 (13.9)	28.6 (20.3)^b^
**Gambling type, n (%)**			
	Bets	2006 (41.10)	69 (46.0)^c^
	Casino	1650 (33.80)^c^	43 (28.7)
	Scratch cards	1043 (21.37)^b^	11 (7.3)
	Poker	592 (12.13)	41 (27.3)^b^
	Lottery	515 (10.55)^c^	10 (6.7)
	Others	182 (3.73)	16 (10.7)^b^
**Media for gambling, n (%)**			
	Solely offline	3419 (70.05)^b^	67 (44.7)
	Web-based or both	1462 (29.95)	83 (55.3)^b^
**Proxies for severity of gambling**			
	Gambling frequency weekly or more, n (%)	4418 (90.51)^c^	130 (86.7)
	Time since gambling initiation (years), mean (SD)	14.1 (9.2)	20.1 (9.7)^c^
	History of suicide attempt, n (%)	110 (2.25)	4 (2.7)^c^
**Contact demand, n (%)**			
	Referral requested	1258 (25.77)^d^	24 (16.0)
	Refusal to be referred	101 (2.07)	6 (4.0)^c^
**Actual referral, n (%)**			
	Referred	2879 (58.98)^c^	78 (52.0)
	Nonreferred	2002 (41.02)	72 (48.0)^c^
Self-exclusion, n (%)		247 (5.06)	9 (6.0)^c^

^a^*P*<.05.

^b^*P*<.001.

^c^Not significant.

^d^*P*<.01.

**Table 4:** Association between the actual referral and the characteristics of the gamblers (a multivariate logistic regression analysis).

Independent variables			Adjusted odds ratio (95% CI)		*P* value	
**Gender**						
	Female			Ref^a^		Ref
	Male			1.12 (0.99-1.26)		.07
**Age (years)**						
	<20			0.85 (0.60-1.20)		.36
	20-29			0.88 (0.75-1.03)		.10
	30-39			Ref		Ref
	40-49			0.96 (0.82-1.13)		.62
	50-59			0.86 (0.72-1.02)		.09
	≥60			0.85 (0.69-1.04)		.11
**Gambling type**						
	**Bets**					<.001
		No		Ref		
		Yes		1.46 (1.27-1.67)		
	**Casino**					<.001
		No		Ref		
		Yes		1.38 (1.21-1.57)		
	**Scratch cards**					<.001
		No		Ref		
		Yes		1.83 (1.58-2.12)		
	**Poker**					<.001
		No		Ref		
		Yes		1.35 (1.14-1.61)		
	**Lottery**					
		No		Ref		Ref
		Yes		1.27 (1.03-1.56)		.03
**Media used for gambling**						
	Solely offline			Ref		Ref
	Web-based and both			0.86 (0.75-0.98)		.03
**Proxies for severity of gambling**						
	**Gambling frequency**					
		Less than a week		Ref		Ref
		Weekly or more		1.73 (1.40-2.15)		<.001
	**History of suicide attempts**					
		No		Ref		Ref
		Yes		2.13 (1.51-3.02)		<.001
**Contact demand**						
	**Referral requested**					
		No		Ref		Ref
		Yes		17.76 (14.92-21.13)		<.001
	**Refusal to be referred**					
		No		Ref		Ref
		Yes		0.35 (0.26-0.49)		<.001

^a^Ref: reference.

## Discussion

### Principal Findings

We included 9474 contacts from gamblers over 5 years, among whom 55.1% (5221/9474) were referred. Our main findings were that real-time chat contacts differed significantly from helpline contacts, as they were younger and more often Web-based gamblers, and that referral was not only associated with gambling severity variables, such as a history of suicide attempts and gambling frequency, but also with Web-based gambling per se. In addition, referral was associated with a demand for referral, but female contacts less often expressed a demand for referral.

### Evolution Over the Study Period

The decrease observed in poker-gambling contacts over the study period could be explained by a decline in the turnover of the legal poker market in France in 2013 [31]. In addition, the decrease in frequency of the other games could correspond to a decrease in gambling not included in the legal offer and thus to a possible regression of illegal gambling. Thus, helplines could be an interesting epidemiological tool for tracking problems related to gambling that follow the commercial offer of gambling activities [28]. On the contrary, the increase in the proportion of contacts engaged solely in offline gambling does not reflect the market, as Web-based gambling is growing fast and seems to cause more problems [29] and to be related to less help seeking [30] than offline gambling. One hypothesis is that the problem gambling prevention campaign, which accompanied the opening of the Web-based gambling market in 2010, may have attracted people who already had a gambling problem and were therefore mostly solely offline gamblers. Communication on the services available for problem gambling was a part of this prevention campaign.

### Gender Differences

Our study highlights gender differences among the contacts made by gamblers. Contacts were more often men, which is consistent with several studies [19,20,24,26] and with the latest epidemiological survey in France, which evidenced 70% of men among problem gamblers [3]. Female gamblers were older, as in several other studies [19,20,22,26]. The types of gambling were different between men and women: men were more often engaged in betting and poker gambling, and women were more often engaged in casino gambling, scratch cards, and lotteries. These differences were also found in several previous studies [19,20,26] on problem gambling and support the quality of our data. Male and female gamblers seemed to present the same levels of severity as assessed with the proxy variables used here, ie, gambling frequency, time since gambling initiation, and history of suicide attempts, supporting other findings where severity was assessed on the Problem Gambling Severity Index [26]. Other studies showed even greater severity among women. However, women were less likely to express a demand for referral. This could be a special situation, with women having as severe a gambling problem as men, seeking help, but more reluctant than men to express a demand for formal guidance. This element suggests that the follow-up of female gamblers could involve a taboo, indirectly fueling the treatment gap. However, even if little information is available about gender differences in barriers to seeking help, men might be more vulnerable than women to shame, embarrassment, pride, or stigma [10].

### Actual Referral

The referral rate in this study was lower than that found in other studies [23]. According to our hypothesis, contacts’ attitudes (ie, referral requested or refusal to be referred) were not the only predictor of being referred. The severity of the pathology as assessed by the two proxies (ie, gambling frequency and history of suicide attempts) was predictor of being referred. We noted that Web-based gamblers were less often referred, although they appear to be the category of gamblers that is increasing most markedly [3,29].

### Helpline Versus Real-Time Chat

It should be noted that despite the spectacular increase in the use of the internet in our lives, the telephone proved to be much more popular (97.0% of contacts between May 2013 and December 2015) when seeking help in the context of problem gambling in France. It is our opinion that the very low level of use of real-time chat compared with the helpline could be because of the lack of publicity for the online service. In the advertising strategy, the only general measure consists in the obligation to display a health message with the phone number of JIS in any commercial communication in favor of a gambling operator. In addition, there is little communication about the internet services offered by JIS. The launch of real-time chat in May 2013 was intended to reach a younger population that is more likely to be using the internet, and it seems that this objective was attained, even though the total number of users of real-time chat was relatively small. Even if its use is increasing, the promotion of the real-time chat service could enable faster growth. Owing to the massive increase in the use of the internet in our lives and the fast development of the Web-based gambling market [3,29], reaching gamblers on the internet will clearly be the challenge for the future. We demonstrated that chat interviews could be particularly suited to young Web-based gamblers. However, this type of online contact should not prevent referral, as helpline and chat contacts presented the same levels of severity assessed with the chosen proxies.

### Limitations

The JIS does not use a screening tool or diagnostic scale for problem gambling, which could have enabled a better clinical characterization of the population. It is highly probable that most gamblers contacting the helpline or real-time chats were problem gamblers, as demonstrated in other studies in similar settings (94% [26], 91% [22], and 82% [23]). By nature, contacts were gamblers who had identified damage related to gambling and felt the need to ask for help. The proxy measures used to assess severity should thus be interpreted with considerable caution.

Contacts were anonymous, but counselors noted that 19.7% of the gamblers mentioned having contacted the service previously (the missing data on this variable were high, at 77.5%, and were not imputed). Thus, there could be a proportion of duplicates among the contacts. This observation should be put into perspective, because each call is anonymous and there is no possible follow-up via JIS, so each contact was considered as a new event. For some patients, the service could be sufficient, as found in one study after a single session, showing improvement in psychological outcomes (ie, increased confidence score and decreased distress) [35], or in another, which observed a decrease in the number of days of gambling and in financial losses [36]. In addition, we have no information of individuals’ attendance in care facilities following referral. The gap between referral and actual attendance could be around 20% according to one study [23]. This raises the importance of not targeting a low referral rate, as, later, attendance for face-to-face appointments would probably be even lower. The JIS structure for problem gambling could benefit from improvements through the development of partnerships with care centers offering face-to-face appointments directly and as quickly as possible, as is the case, eg, in Australia [26].

### Clinical Implications

In the literature, recourse to gambling helplines is often a first contact. In 2001, the majority of gamblers using the New England helpline were seeking help for the first time [19]. Similarly, in 2011, about 90% of people using the West Virginia Problem Gamblers Help Network had never previously sought help for problem gambling [23]. The use of this kind of service could serve as a catalyst and enable people to access face-to-face care facilities. Thus, 55% of people using the West Virginia Problem Gamblers Help Network attended a face-to-face assessment interview [23]. More than 90% of participants on the Gambling Help Online website between November 2010 and February 2012 accessed further help from formal or nonformal support facilities following a helpline contact [35]. The use of new technologies, such as real-time chats, could help to remove some of the barriers to treatment encountered by gamblers [37] and could be a valuable tool to prevent early complications of gambling addiction, such as spiraling social costs and suicidal tendencies. Reasons reported by participants who had completed an online counseling session for problem gambling on the Gambling Help Online website between November 2010 and February 2012 fell into four categories: confidentiality and anonymity, convenience and accessibility, service system access, and a preference for the therapeutic medium [38]. With the development of internet-based therapy, JIS could be the tool of choice to treat a population that cannot or will not visit a face-to-face specialized care facility.

### Conclusions

This is the first study describing contacts to a national helpline and chat service for problem gambling in France. The referral rate was low and linked to gamblers’ demands and the severity of problem gambling. It was also lower among Web-based gamblers. Female gamblers less often expressed a demand for referral, and an effort could be made to facilitate communication among female gamblers and to detect their needs to help them more effectively. The real-time chat mode seems to respond to the need to broaden the media offer for help, reaching a younger population of Web-based gamblers. However, it is underused, and its development is a major challenge for the future. Overall, increasing the referral rate after initial non–face-to-face contact is a public health issue, and our study provides avenues for improving the referral rate.

## Appendix

Multimedia Appendix 1 Monthly time series (2011-2015, except for (12) May 2013-December 2015), trends, and regression lines for characteristics of Joueurs Info Service contacts from gamblers: (1) Proportion of women; (2) Mean age of contacts; (3) Mean duration of contacts; (4) Proportion of bets; (5) Proportion of casino games; (6) Proportion of scratch cards; (7) Proportion of poker; (8) Proportion of lottery; (9) Proportion of other games; (10) Proportion of solely offline gamblers; (11) Proportion of weekly gamblers; (12) Proportion of real-time chat users.

## Abbreviations

ADALIS: Addictions Drogues Alcool Info Service
aOR: adjusted odds ratio
JIS: Joueurs Info Service
MAR: missing at random
