# Supplementary figures and images for: Characteristics of Gamblers Who Use the French National Problem Gambling Helpline and Real-Time Chat Facility: Longitudinal Observational Study
Source: JMIR Form Res. 2020 May 20;4(5):e13388. doi: 10.2196/13388 (PMC7270843; doi:10.2196/13388)

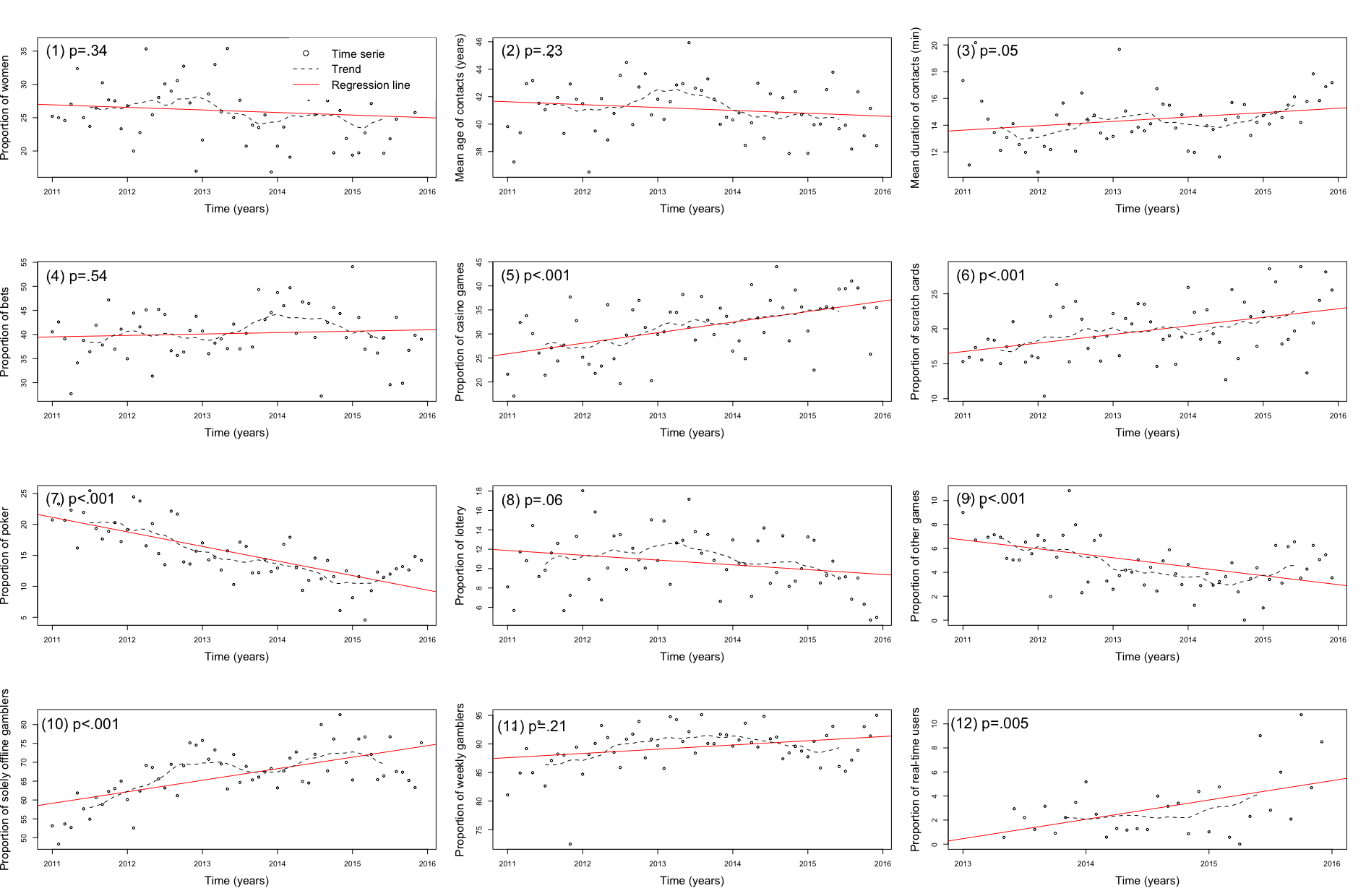

Supplement: Multimedia Appendix 1 [file formative_v4i5e13388_app1.png]
